# Molecular Biomarkers: Tools of Medicine

**DOI:** 10.1155/2013/595496

**Published:** 2013-11-27

**Authors:** Prabir K. Mandal, Shivani Soni, R. Renee Reams, Tiziano Verri, Anita Mandal, Sudhish Mishra

**Affiliations:** ^1^Department of Biology, Edward Waters College, 1658 Kings Road, Jacksonville, FL 32209, USA; ^2^Department of Biological Sciences, Alabama State University, Room No. 325, Life Science Building 1627, Hall Street, Montgomery, AL 36104, USA; ^3^College of Pharmacy & Pharmaceutical Sciences, Florida A&M University, 1520 M L King Boulevard, Tallahassee, FL 32307, USA; ^4^Laboratory of General Physiology, Department of Biological and Environmental Sciences and Technologies, University of Salento, I-73100 Lecce, Italy; ^5^Department of Translational Science and Molecular Medicine, MSU College of Human Medicine and Van Andel Institute, 333 Bostwick Avenue NE, Grand Rapids, MI 49503, USA

Molecular biomarkers are emerging as the key indices for the management of patients with significant diseases. Motivated by the systematic effort to define the human genome, the creation of rapid analytic technologies for evaluating nucleic acids and proteins has provided the technological “boom” for the development of molecular biomarkers. Collaboration and cooperation between stakeholders involved in biomarker development, application, and regulation may be the most expeditious route toward the translation of laboratory discovery into patient management. In summary, intensive research has originated multiple factors or biomarkers that are likely to be helpful in diagnosis, characterization, and therapy selection of different patients. A thorough understanding of the relevance of each biomarker will be the key to efficiently diagnose a disease and direct the patients towards the drugs more likely to be of benefit based on their particular profile. 

The papers selected for this special issue represent an excellent panel for addressing the molecular biomarkers as the future tools of medicine. This special issue contains thirteen papers.

In the paper entitled “*Circulating microRNAs and kallikreins before and after radical prostatectomy: are they really prostate cancer markers?*,” M. G. Egidi et al. presented 38 patients with prostate cancer. They suggested that two miRNAs (miR-21 and miR-141) could be involved in post surgical inflammatory processes. Postoperative serum kallikreins showed a significant decrease, highlighting the potential usefulness of kallikreins apart from PSA as potential prostate cancer markers.

In the paper entitled “*A novel differential predict model based on matrixx-assisted laser ionization time of flight mass spectrometry and serum ferritin for acute graft-versus-host disease*,”. C.-Y. Zhang et al. investigated the possibility of pre warning the risk of an acute graft-versus-host disease (aGVHD) before and after allogeneic hematopoietic stem cell transplantation (allo-HSCT) by serum profiling combining with serum ferritinin. Their joint prewarning model could predict the risk of aGVHD, especially severe aGVHD before transplant which provide a reliable method to continuously monitor the condition of patients.

In the paper entitled “*The use of multidimensional data to identify the molecular biomarker for pancreatic ductal adenocarcinoma*,” L. Zhuang et al. presented that they have adopted an integrative approach to simultaneously identify biomarker and generate testable hypothesis from multidimensional omics data. They have found that PER2 expression was highly associated with the survival data, thus representing a novel biomarker for earlier detection of pancreatic ductal adenocarcinoma (PDAC).

In the paper entitled “*Clinical evaluation and cost-effectiveness analysis of serum tumor markers in lung cancer*,” R. Wang et al. showed that combinations of four tumor markers (SCCA, NSE, CEA, and CYFRA21-1) improved the sensitivity for lung cancer and different combination panels had their own usefulness. NSE, CEA, and CYFRA21-1 were the optimal combination panel with highest Youden's index (0.64), higher sensitivity (75.76%), and specificity (88.57%), which can aid in clinical diagnosis of lung cancer.

In the paper entitled “*Immune parameters in the prognosis and therapy monitoring of cutaneous melanoma patients: experience, role, and limitations*,” M. Neagu et al. reported the follow-up for 36 months of the immune parameters of patients diagnosed in stages I–IV, namely, pre- and postsurgery immune circulating peripheral cells and circulating intercommunicating cytokines.

In the paper entitled “*Comparative gene expression profiling in human cumulus cells according to ovarian gonadotropin treatments*,” S. Assou et al. provided an exclusive study characterizing gene expression profiles in cumulus cells (CCs) of periovulatory follicles from patients undergoing HP-hMG and rFSH gonadotropin treatments during *in vitro* fertilization cycles. This project has characterized the expression of these genes as biomarkers of *in vitro* embryo quality.

In the paper entitled “*Aptamers: novel molecules as diagnostic markers in bacterial and viral infections?*,” F. M. Zimbres et al. urged an urgent need to discover novel diagnostic as well as therapeutic tools against infectious agents. They viewed that the systematic evolution of ligands by exponential enrichment (SELEX) represents a powerful technology to target selective pathogenic factors as well as entire bacteria or viruses. SELEX uses a large combinatorial oligonucleic acid library (DNA or RNA) which is processed by a high-flux *in vitro* screen of iterative cycles.

In the paper entitled “*Distribution of ABO blood group and major cardiovascular risk factors with coronary heart disease*,” S. Biswas et al. viewed that the AB blood group decreases the risk of CHD in healthy controls; it might be due to the higher concentration of high density lipoprotein cholesterol (HDL-c), while the O blood group increases the risk of CHD due to lower HDL-c levels in Bengali population of eastern part of India.

In the paper entitled “*Immunomodulatory effect of continuous veno-venous hemofiltration during sepsis: preliminary data*,” G. Servillo et al. reported that severe sepsis and septic shock are the primary causes of multiple organ dysfunction syndrome (MODS), which is the most frequent cause of death in intensive care unit patients. Many pro- and anti-inflammatory mediators, such as interleukin-6 (IL-6), play a key role in septic syndrome. Continuous renal replacement therapy (CRRT) removes in a nonselective way pro- and anti-inflammatory mediators. The authors investigate the effects of continuous venovenous hemofiltration (CVVH) as immunomodulatory treatment of sepsis in a prospective clinical study.

In the paper entitled “*Acetylcholinesterase as biomarker in environmental and occupational medicine: new insights and future perspectives*,” M. G. Lionetto et al. viewed that Acetylcholinesterase (AChE) is a key enzyme in the nervous system (NS), since it terminates nerve impulses by catalyzing the hydrolysis of acetylcholine. As a specific molecular target of organophosphate and carbamate pesticides, AChE activity and inhibition are a human biological marker of pesticide poisoning. Thus, it is used to study the effects of the exposure to organophosphate and carbamate pesticides on NS in occupational and environmental medicine. This paper reviews and discusses the recent findings about AChE, including its sensitivity to other pollutants and expression of different splice variants. These insights open new perspectives for the use of this biomarker in environmental and occupational human health monitoring.

In the paper entitled “*Polyisoprenylated methylated protein methyl esterase is both sensitive to curcumin and overexpressed in colorectal cancer: implications for chemoprevention and treatment*,” F. Amissah et al. discussed polyisoprenylated methylated protein methyl esterase (PMPMEase) which cocatalyzes the polyisoprenylation pathway required to process various monomeric G proteins. Mutation of these G proteins is considered to be responsible for 50% of colorectal cancers. This interesting finding suggests that elevated PMPMEase activity and its overexpression can be one of the candidate markers for early diagnosis of colorectal cancer. Susceptibility of this enzyme to curcumin also suggests that PMPMEase can be a potential candidate for targeted anticancer therapy.

In the paper, entitled “*Emerging therapeutic biomarkers in endometrial cancer*,” P. Dong et al. reviewed the current status of molecular therapies tested in clinical trials and mainly discussed the potential therapeutic candidates that are possibly used to develop more effective and specific therapies against endometrial cancer progression and metastasis.

In the paper entitled “*Mouse prostate epithelial luminal cells lineages originate in the basal layer where the primitive stem/early progenitor cells reside: implications for identifying prostate cancer stem cells*” J. Zhou et al. have developed an *in vivo* cell fate tracing mouse model and an *in vivo* slow-cycling cell label mouse model to provide further insight into this question. Through genetic manipulation in the animals, their findings indicate that the basal cell lineage can produce more differentiated luminal cells; the putative mouse prostate stem cells (which are slow-cycling and responsible for tissue maintenance) likely reside in the basal layer.

 Though the selected topics and papers are not an exhaustive representation of the entire area of molecular biomarkers and tools of medicine, yet they represent the rich and many-faceted knowledge that we have the privilege of sharing with the readers. 

